# Supplementary material for: Towards extending the aircraft flight envelope by mitigating transonic airfoil buffet
Source: Nat Commun. 2024 Jun 12;15:5020. doi: 10.1038/s41467-024-49361-3 (PMC11169508; doi:10.1038/s41467-024-49361-3)
Supplement: Supplementary file 1 — Supplementary Information [file 41467_2024_49361_MOESM1_ESM.pdf]

Supplementary Information: Towards extending the aircraft flight envelope by mitigating transonic airfoil buffet

Esther Lagemann[†1,2], Steven L. Brunton[1], Wolfgang Schröder[2], and Christian Lagemann[†1,2]

[1]AI Institute in Dynamic Systems, Department of Mechanical Engineering, University of Washington, Seattle, U.S.
[2]Institute of Aerodynamics, RWTH Aachen University, Wüllnerstraße 5a, 52062 Aachen, Germany

[†]Authors contributed equally.

1 Supplementary section: Experimental data and post-processing

The experimental setup, i.e., the simultaneous Particle-Image Velocimetry (PIV) and Background-Oriented Schlieren (BOS) measurements, includes two cameras per measurement technique as indicated in figure 2 of the main paper. This solution was chosen to increase the spatial resolution of the acquired flow quantities compared to a single camera per measurement technique.

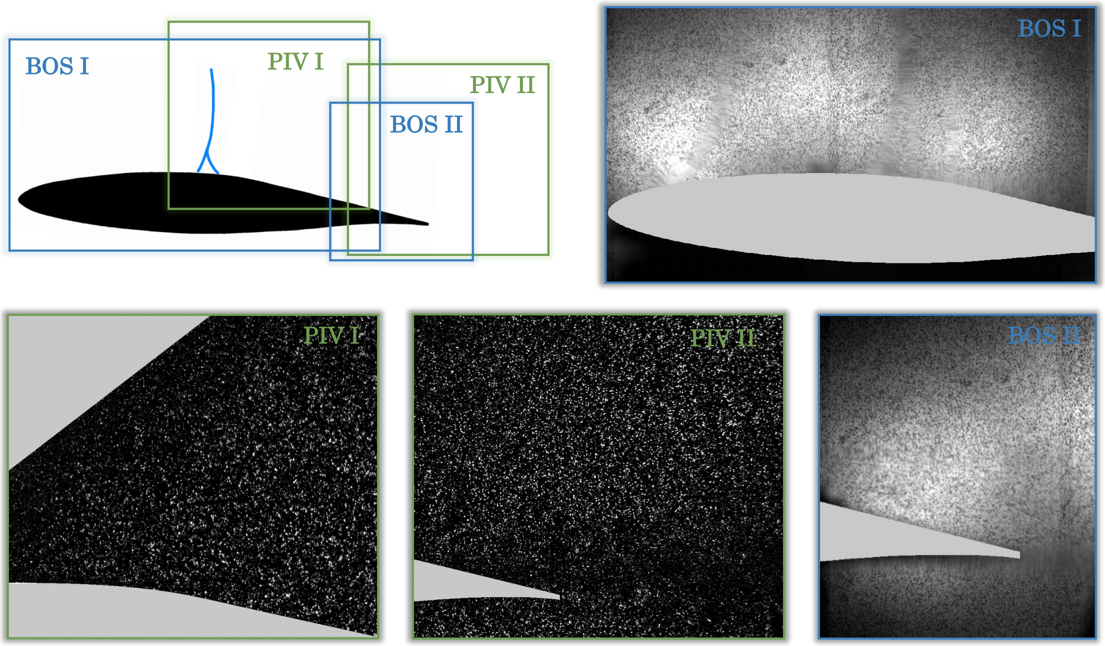

Figure S1: **Raw PIV and BOS images of the reference geometry at Ma = 0.76.** The sketch in the upper left corner shows the field of views captured by each camera with respect to the airfoil. The raw PIV images (lower left, lower center) depict the first exposure of a representative particle image pair with a moving-mean based background subtraction to remove artifacts like light sheet reflections. The BOS images (upper and lower right) are instantaneous snapshots, which are compared to the background dot pattern at zero flow to calculate the density gradient fields. Image parts without meaningful information are either masked, e.g., the upper left triangular-shaped region in ”PIV I”, or cropped, e.g., the lower image part in ”BOS I”. Source data are provided as a Source Data file.

Figure S1 depicts the field of views captured by each camera with respect to the airfoil (upper left) and examples of raw images for each camera. The PIV setup focuses on the shock wave and the flow downstream of the shock wave. Due to accessibility constraints of the wind tunnel facility, the laser light sheet is not able to illuminate the flow in the leading edge region. This also results in the upper left triangular-shaped region in the "PIV I" image (lower left in figure S1), where no flow field information is available. Similarly, the mounting of the airfoil covers the optical access of the BOS setup with respect to a large region of the airfoil's pressure side.

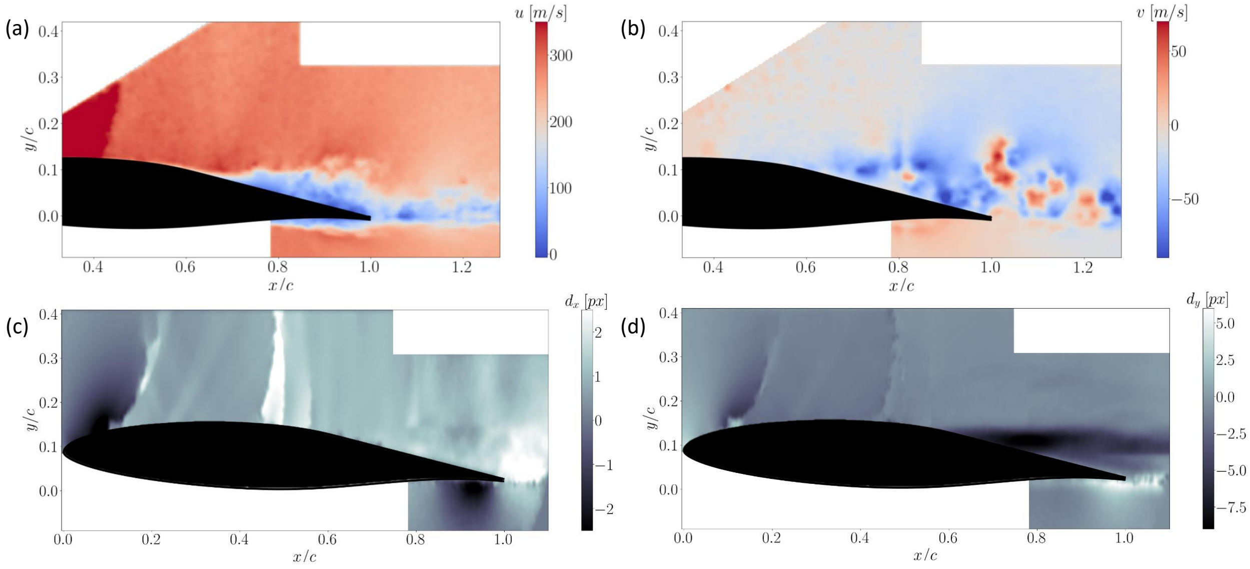

Figure S2: **Instantaneous velocity and density gradient fields of the reference geometry at Ma = 0.76.** (a) streamwise velocity field u, (b) vertical velocity field v, (c) streamwise density gradient field d_x, (d) vertical density gradient field d_y as a function of streamwise x and vertical y location normalized by the chord length c. Please note that velocity values are given in m/s, whereas density gradients values are provided in px, i.e., the displacement fields are shown. A calibration of the density values into physical units is performed when integrating the gradient fields, which is described in section 2.1. Source data are provided as a Source Data file.

Examples of instantaneous velocity (a,b) and density gradient (c,d) fields are provided in figure S2. In contrast to figure S1, the flow field information of both cameras is combined into a single image. For the overlapping region, a weighted interpolation is used.
For determining the shock wave position within each snapshot, the streamwise density gradient fields are pre-processed with a 2D NA-MEMD as described in section 4.2 (*Methods, Data evaluation*). The shock wave profile distinctly appears in the first mode, which allows an accurate extraction of the shock wave position for each individual time instant as exemplified in figure S3 (a,b). For all locations encompassed by the shock profile contour, the weighted mean is used as the (averaged) shock position within each snapshot. An extract of the time-varying shock wave location extracted from the original density gradient field vs. the pre-processed field is provided in figure S3 (c).

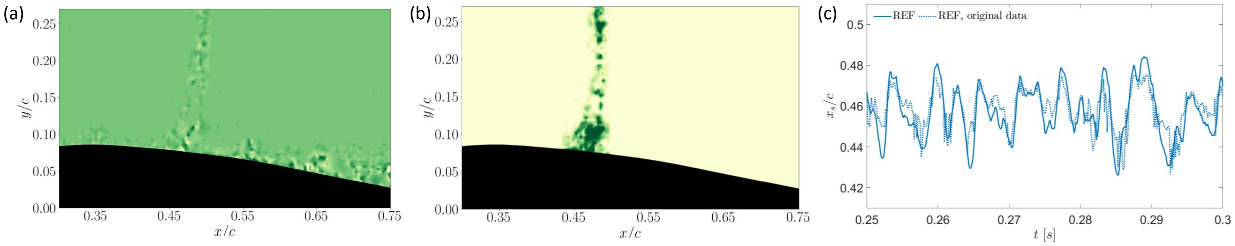

Figure S3: **Shock position detection.** (a) shows an extract of the streamwise density gradient around the shock wave for an arbitrary time step of the reference configuration. (b) provides the same data extract but pre-processed based on the 2D NA-MEMD (first mode is selected) to enhance the shock detection. (c) shows the time-dependent shock wave position obtained by using the unmodified density gradient fields and the pre-processed fields. The pre-processed data exhibits less fluctuations resulting from the more distinct detection of the shock wave. Source data are provided as a Source Data file.

2 Supplementary section: Deriving the aerodynamic performance from BOS and PIV data

The following paragraph is taken from the *Methods* section of the main paper and repeated here as an introduction to the more detailed description of the workflow.

The aerodynamic performance of the airfoil is derived from the flow field measurements, i.e., the BOS and PIV data. Once the pressure field is known, the surface pressure can be integrated along the airfoil contour to provide a reasonable approximation for the lift and the drag values. The pressure field is derived in three steps. First, the density gradient fields extracted via the BOS measurements are integrated with a weighted least-squares optimization methodology [1] yielding the density field (section 2.1). Second, the temperature field is determined using the velocity data from the PIV measurements in combination with the energy equation (section 2.2). Third, the ideal gas law is applied to calculate the pressure field from the density and the temperature field (section 2.3). In contrast to other studies estimating the pressure field and the aerodynamic performance from PIV data of the compressible flow around an airfoil [2, 3], we additionally invoke the density information from the BOS measurements. This allows the derivation of the quantities of interest with a higher accuracy involving less assumptions about the flow state.

Since the BOS and the PIV data have a different spatial resolution, the BOS based flow quantities are interpolated onto the same grid as the PIV data. To calculate the density, temperature, and pressure fields, we use the time-averaged velocity and density gradient fields. Since our approach relies on a few assumptions, e.g., ideal gas properties and isentropic flow upstream and downstream of the shock wave, respectively, we expect our results to be physically significant in a statistical sense. In fact, we are able to verify the accuracy of the time-averaged pressure data calculated for the reference configuration, i.e., with an ordinary trailing edge, at a Mach number of $\mathrm{Ma} = 0.73$ and an angle of attack of $\alpha = 3.5°$ with pressure tap measurements [4], which have been conducted for the same airfoil in the same wind tunnel facility. The good agreement shows that our approach provides physically meaningful pressure information (section 2.4).

Since our BOS and PIV measurements do not capture the entire airfoil, we cannot calculate the overall lift and drag values since we do not have pressure information at the entire airfoil surface. However, we have pressure data in the regions where we expect the highest alteration due to the porous trailing edges. That is, downstream of the shock wave on the suction side and along the porous trailing edge on the pressure side. Therefore, we calculate how the contributions of these regions to the overall drag and lift values change in the presence of porous trailing edges. This convincingly shows the governing trends of the aerodynamic performance. As a reference, the complete pressure data provided in [4] is used to estimate drag and lift

values for the entire airfoil profile. Subsequently, the lift and drag coefficients based on the restricted area of the present study are calculated and compared between a pressure tap based and a flow field based analysis (sections 2.6, 2.7). In the following, we provide detailed information about the derivation of the pressure fields and the aerodynamic forces.

2.1 Supplementary subsection: Density field calculation from BOS data

The BOS measurements provide density gradient fields, which are shown in figure S2 (c,d) for an arbitrary time step. The density gradient fields are integrated to yield the respective density information using the weighted least-squares optimization methodology proposed in [1]. This method requires Dirichlet boundary conditions, i.e., density values at the image boundaries. The first value is imposed at the inflow boundary, where we derive the density ρ_∞ from the isentropic relation for an ideal gas from equation 1.

$$\rho_\infty = \rho_{01} \left(\frac{T_\infty}{T_0} \right)^{\frac{1}{\gamma - 1}} \tag{1}$$

$$T_\infty = \frac{T_0}{1 + \frac{\gamma-1}{2} \mathrm{Ma}_\infty^2} \tag{2}$$

The freestream temperature T_∞ can be calculated by the energy equation using the known freestream Mach number Ma_∞. Since the inflow in a certain distance to the airfoil model is approximately steady, quasi-1D, adiabatic, and inviscid, the simplified energy equation given in equation 2 reliably approximates the flow state. The remaining variables are isentropic stagnation state values, which are known from the temperature measured in the stagnation chamber of the wind tunnel ($T_0 = 288.15\ K$) and the atmospheric conditions ($\rho_{01} = 1.225\ kg/m^3$, $p_0 = 101\ 325\ Pa$). The heat capacity ratio is $\gamma = 1.402$.
The remaining boundary conditions are set at the upper image boundary ρ_I and at the outflow boundary ρ_{II} (equation 3). They require additional flow field information, which are derived from the PIV measurements. The stagnation density ratio ρ_{02}/ρ_{01} across the shock wave (1: upstream, 2: downstream) is given by the Pitot-Rayleigh relation for a normal shock wave (equation 4). This relation is given for the stagnation pressure ratio across the shock wave. However, the constant stagnation temperature T_0 across the shock allows us to replace the pressure ratio by the density ratio assuming ideal gas behavior. The only additional flow quantity required to solve for the downstream stagnation density ρ_{02} is the Mach number upstream of the shock Ma_1, which is determined using the PIV data. The calculation of Ma_1 (equation 5) as well as the isentropic relation (equation 3) require temperature information ($T_1, T_{I,II}$), which we derive from the PIV data in combination with the energy equation (equation 6). The specific gas constant is set to $R = 287.05\ J/kgK$. Similar to the inflow boundary condition, we assume that the flow in a certain distance to the airfoil model and downstream of the shock wave is quasi 1D, the shock wave is normal, and the flow exhibits isentropic behavior under the ideal gas law. Influences of the wind tunnel's upper and lower walls are negligible since their contour is adjusted to match the streamlines as described in [4, 5].

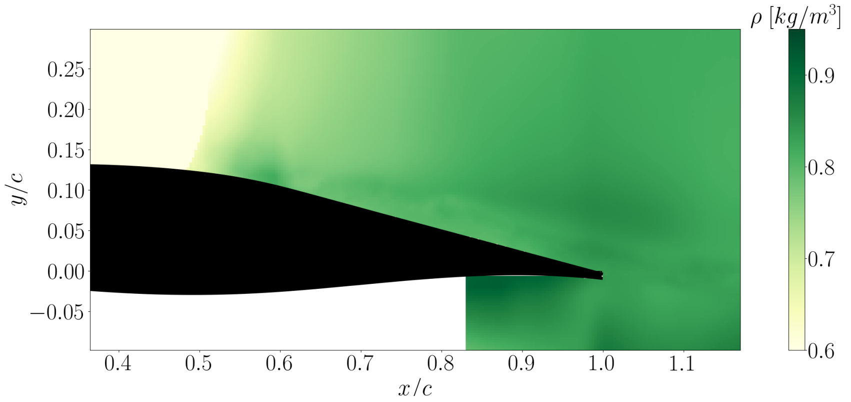

Figure S4: **Time-averaged density field at Ma = 0.73.** The density field ρ is determined by applying a weighted least-squares optimization algorithm [1] to the density gradient fields obtained from the BOS measurements. Source data are provided as a Source Data file.

$$\rho_{I,II} = \rho_{02} \left(\frac{T_{I,II}}{T_0} \right)^{\frac{1}{\gamma-1}} \tag{3}$$

$$\rho_{02} = \rho_{01} \left[1 + \frac{2\gamma}{\gamma+1} \left(\mathrm{Ma}_1^2 - 1 \right) \right]^{-\frac{1}{\gamma-1}} \left[\frac{(\gamma+1)\,\mathrm{Ma}_1^2}{2+(\gamma-1)\,\mathrm{Ma}_1^2} \right]^{\frac{\gamma}{\gamma-1}} \tag{4}$$

$$\mathrm{Ma}_1 = \frac{u_1}{\sqrt{\gamma R T_1}} \tag{5}$$

$$T_{1,I,II} = T_0 - \frac{\gamma-1}{\gamma R} \frac{u_{1,I,II}^2}{2} \tag{6}$$

In figure S4, the time-averaged density field at Ma = 0.73 is shown. Please note that only the part of the density field is shown for which complementary PIV data exists. On the pressure side of the airfoil, only limited PIV data are available since the laser light sheet is blocked by the airfoil model.

2.2 Supplementary subsection: Temperature field calculation using PIV data

The temperature field is derived from the velocity field determined by the PIV data. Figure S2 (a,b) shows the velocity fields of an arbitrary time step for reference. By exploiting the energy equation 7, the respective temperature field is obtained. The time-averaged field is given in figure S5.

$$T = T_0 - \frac{\gamma-1}{\gamma R} \frac{u^2}{2} \tag{7}$$

Using this form of the energy equation assumes steady, inviscid, and quasi-1D flow with ideal gas properties. By applying this equation to the time-averaged instead of the instantaneous flow field, we minimize the uncertainty associated with these simplifications. Nevertheless, the flow in the separated boundary layer is neither 1D nor inviscid such that the resulting temperature field only approximates the true temperature distribution.

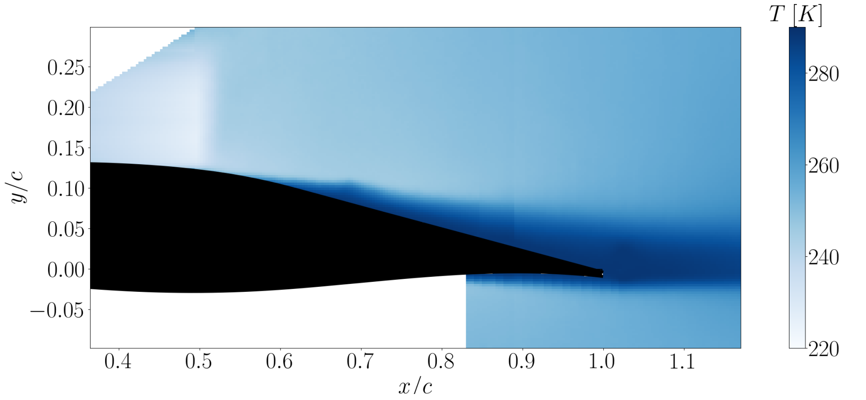

Figure S5: **Time-averaged temperature field at Ma $= 0.73$.** The temperature field T is calculated using the energy equation and the velocity field provided by the PIV data. Source data are provided as a Source Data file.

2.3 Supplementary subsection: Pressure field calculation

Once the density and the temperature fields are calculated, the ideal gas law $p = \rho RT$ provides the corresponding pressure field. The time-averaged result is shown in figure S6.

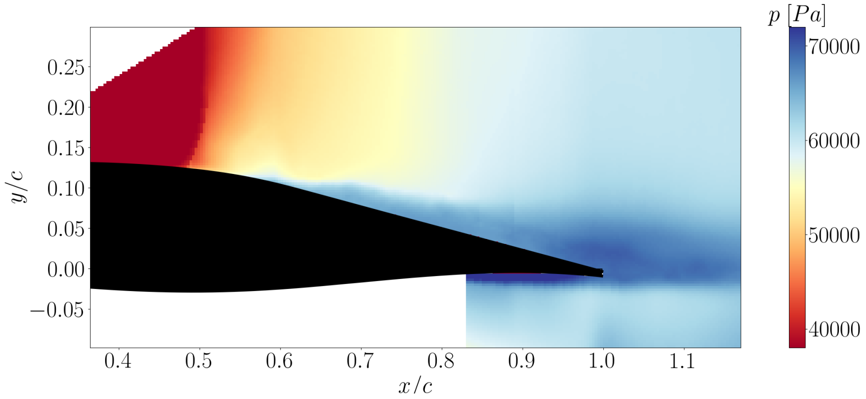

Figure S6: **Time-averaged pressure field at Ma $= 0.73$.** The pressure field p is obtained via the ideal gas law. The density is derived by integrating the density gradient fields from the BOS data (section 2.1) and the temperature field is based on the energy equation and the velocity field from the PIV data (section 2.2). Source data are provided as a Source Data file.

2.4 Supplementary subsection: Validation of the pressure field derived from BOS and PIV data

To verify the derived pressure field, the time-averaged pressure data at Ma $= 0.73$ are compared with surface pressure measurements reported in [4]. The authors conducted similar measurements at Ma $= 0.73$ and an angle of attack of $\alpha = 3.5°$ in the same wind tunnel facility using the same supercritical airfoil. However, their model was equipped with pressure taps along the suction and the pressure side whose locations are defined in figure 4 in [4]. The measurements were used to calculate the time-averaged distribution of the pressure coefficient (figure 7 in [4]). In figure S7, we compare this distribution with the pressure coefficients

of the present study, which are calculated using the pressure values in the direct vicinity of the airfoil surface. The pressure coefficient reads

$$c_p = \frac{p - p_\infty}{0.5\rho_\infty u_\infty^2} = \frac{2}{\mathrm{Ma}_\infty^2 \gamma}\left(\frac{p}{p_0}\left(1 + \frac{\gamma - 1}{2}\mathrm{Ma}_\infty^2\right)^{\frac{\gamma}{\gamma-1}} - 1\right) \quad (8)$$

This derivation invokes the same assumptions that were applied in the previous calculations, i.e., ideal gas behavior and 1D, steady, inviscid, and isentropic flow.

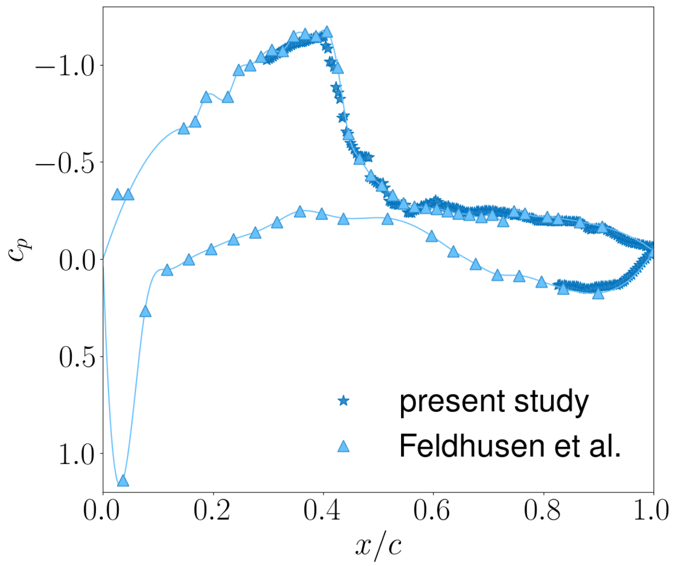

Figure S7: **Pressure coefficients at Ma = 0.73.** The pressure coefficients c_p provided by Feldhusen et al. [4] are obtained from surface pressure sensors, while the values of the present study are derived from the BOS and PIV measurements. Both studies are conducted with an identical airfoil at the same freestream conditions in the same wind tunnel facility. Source data are provided as a Source Data file.

Figure S7 shows good agreement between the pressure measurements and the derived pressure data. The deviations are largest around the shock wave, which is justifiable considering the aforementioned assumptions. Since we expect the most significant flow field changes induced by the porous trailing edges further downstream of the shock wave, this uncertainty in the pressure distribution across the shock wave is acceptable for the purpose of the present study, where we are primarily interested in the trends of the aerodynamic performance. A similar conclusion holds for the missing data in the leading edge region. Since flow field information cannot travel upstream in supersonic flow, the flow topology upstream of the shock wave on the suction side does not change significantly. It can be affected primarily by the alteration of the shock wave position itself. Since the porous trailing edges shift the shock wave by only 1.0 % and 2.1 %, respectively, we do not expect a significant influence on the upstream flow field.

In summary, the current comparison of the pressure field data shows the satisfactory quality of the determination of the pressure distribution from the BOS and PIV data.

2.5 Supplementary subsection: Density, temperature, and pressure fields in the presence of porous trailing edges

Figure S8 shows the time-averaged density, temperature, and pressure fields for the configurations of interest in the present study, i.e., the reference configuration and both porous trailing edges at Ma $= 0.76$. They are derived using the same approach outlined in the previous sections for the lower Mach number case.

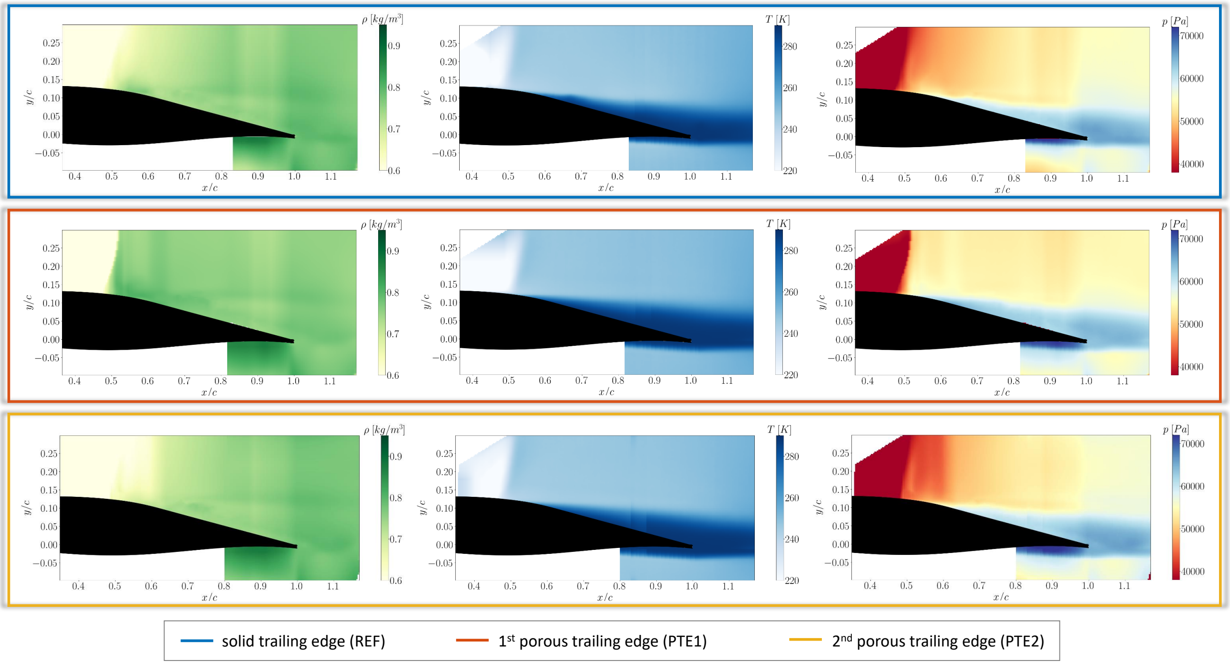

Figure S8: **Density ρ, temperature T, and pressure p fields at Ma $=$ 0.76.** Source data are provided as a Source Data file.

2.6 Supplementary subsection: Lift and drag calculation

The majority of the following paragraph is already described in the *Methods* section of the main paper and repeated here to provide a continuous description of the workflow used to derive the aerodynamic quantities. The aerodynamic force and thus, the lift and drag values, are obtained by integrating the surface pressure along the airfoil. Due to the two-dimensional nature of the airfoil, the force F is calculated per unit depth such that the area used for the pressure integration reduces to the airfoil perimeter S (equation 9). Since we only have discrete pressure values, we use a Riemann sum to approximate the integral. In this case, the local surface increment $\mathrm{d}s_i$ is tangential to the airfoil surface since the pressure acts perpendicular to the surface. The increment $\mathrm{d}s_i$ is obtained from a piecewise linear interpolation of the profile coordinates. To obtain the horizontal and the vertical force components, i.e., the drag D and lift L contributions, respectively, we apply equations 10, 11. The angle α is location dependent and can directly be determined from the profile coordinates (again, using a piecewise interpolation). The surface normal vector $\vec{n} = (n_x, n_y)$ accounts for the sign of the respective quantity. Subsequently, all contributions are summed up to yield the overall drag and lift values. Based on equation 12, the lift and drag coefficients are calculated using the freestream quantities. Please note that viscous stresses are neglected for the calculation of the lift and drag since their contribution is negligibly small compared to the pressure effects [2].

$$F = \int_S p \, d\vec{s} \approx \sum F_i = \sum_S p_i \, ds_i \quad (9)$$

$$D = \sum_S F_i \cdot \sin \alpha_i \cdot n_{x,i} \quad (10)$$

$$L = \sum_S F_i \cdot \cos \alpha_i \cdot n_{y,i} \quad (11)$$

$$c_f = \frac{f}{0.5 \rho_\infty u_\infty^2 S} \quad \text{with } f = L, D \quad (12)$$

As previously mentioned, the experimental setup of the present study does not capture the whole airfoil model. Therefore, we are not able to calculate the overall lift and drag values, since this would require flow field information around the entire airfoil. However, our setup captures the areas in which we expect the greatest changes of the flow field due to the installation of the porous trailing edges. Thus, we can assess how the contributions to the total lift and drag forces change in the area of interest. This gives a good indication how the aerodynamic performance of the airfoil is altered in the presence of porous trailing edges.

2.7 Supplementary subsection: Validation of the derived lift and drag contributions

Similar to the pressure data, we verify our lift and drag calculations based on the Ma = 0.73 test case, since we can use the reported pressure coefficients based on pressure tap measurements [4] for comparison. Rearranging equation 8 yields the pressure distribution from the provided pressure coefficients (equation 13), which are subsequently integrated along the airfoil contour as described in the previous section.

$$p = \frac{p_0 \left(c_p \frac{\gamma \mathrm{Ma}_\infty^2}{2} + 1 \right)}{\left(\frac{\gamma - 1}{2} \mathrm{Ma}_\infty^2 + 1 \right)^{\frac{\gamma}{\gamma - 1}}} \quad (13)$$

To increase the accuracy of the estimated aerodynamic forces, the pressure information is interpolated onto a finer discretization using a cubic spline interpolator. In this case, the approximation of the surface tangential increments by a local linear interpolation, which are used for the Riemann sum, is much more accurate. In figure S9, the original pressure coefficients are shown together with the interpolated values. Note that the pressure sensors are only sparsely distributed in the leading edge region since this area was not of interest in the study by Feldhusen et al. [4]. This introduces an uncertainty of the resulting aerodynamic forces in the leading edge region.

As previously mentioned, the present measurement setup does not capture the entire airfoil, which inhibits an estimation of the overall lift and drag forces. Instead, only the contributions to these forces in the covered regions are studied. Nevertheless, we expect the most significant modifications of the flow field due to the implementation of porous trailing edges in these areas, i.e., downstream of the shock wave and in the vicinity of the trailing edge. Thus, the results of this analysis provide a reasonable indication of the trends in the aerodynamic quantities.

In table S1, the respective lift and drag coefficients are presented. We additionally separate the contributions to the lift and drag forces in the rear part ($(\cdot)_{\mathrm{rear}}$), i.e., the area captured by the present measurement setup, based on the pressure ($(\cdot)_{\mathrm{pre}}$) and the suction side ($(\cdot)_{\mathrm{suc}}$). Since the signs of the lift and drag contributions in the trailing edge region are opposite for the pressure and the suction sides, they might cancel each other

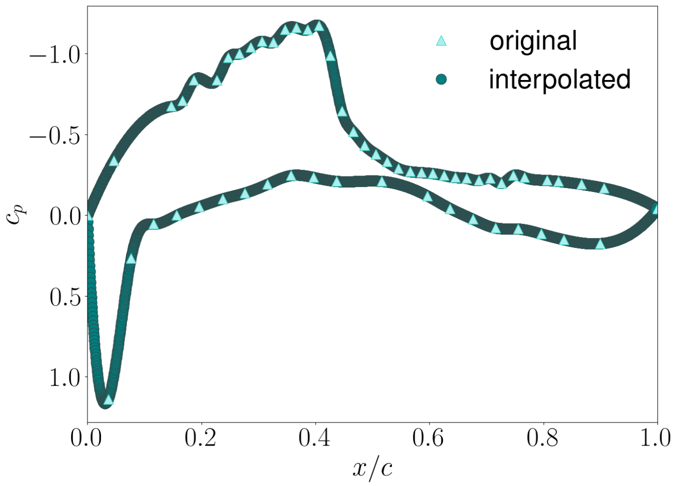

Figure S9: **Pressure coefficients c_p at Ma = 0.73 based on Feldhusen et al. [4].** The original surface pressure measurements are interpolated using a cubic spline interpolator to increase the reliability of the aerodynamic forces. Source data are provided as a Source Data file.

out and provide only limited insight into the aerodynamic behavior. Therefore, we also consider the individual contributions of each airfoil side. This becomes even more interesting for the porous trailing edge configurations to assess how the different airfoil sides are affected.

Table S1: **Lift c_l and drag c_d coefficients at Ma = 0.73.** $(\cdot)_{rear}$: contribution of the rear part of the airfoil that is captured by the present measurement setup. $(\cdot)_{suc}$: contribution of the suction side $(\cdot)_{pre}$: contribution of the pressure side.

	c_l	c_d	$c_{l,rear}$	$c_{d,rear}$	$c_{l,rear,suc}$	$c_{d,rear,suc}$	$c_{l,rear,pre}$	$c_{d,rear,pre}$
Feldhusen et al. [4]	0.254	0.064	0.601	0.313	−2.210	0.414	2.811	−0.101
present study	–	–	0.567	0.315	−2.220	0.414	2.787	−0.100

The lift and drag coefficients obtained from our flow field measurements match the data based on the pressure sensor measurements quite well (table S1). Thus, we conclude that our approach for deriving the aerodynamic quantities in the rear part of the airfoil is reasonably accurate and can be used to assess how the aerodynamic performance changes in the region of highest significance in the presence of porous trailing edges.

2.8 Supplementary subsection: Lift and drag contributions in the presence of porous trailing edges

In table S2, the contributions to the lift and drag coefficients are given for the reference flow and both porous trailing edge designs at Ma = 0.76. Most significantly, PTE1 reduces the lift and increases the drag coefficient, while PTE2 affects the aerodynamic performance only slightly. With respect to the lift coefficient, both, the suction and the pressure side, are affected by PTE1. Regarding the drag force, the primary contribution to the drag increase stems from the suction side and is probably caused by the substantial boundary

layer thickening induced by PTE1.

Table S2: **Lift c_l and drag c_d coefficients at Ma $= 0.76$.** $(\cdot)_{\text{rear}}$: contribution of the rear part of the airfoil that is captured by the present measurement setup. $(\cdot)_{\text{suc}}$: contribution of the suction side $(\cdot)_{\text{pre}}$: contribution of the pressure side.

	$c_{l,\text{rear}}$	$c_{d,\text{rear}}$	$c_{l,\text{rear,suc}}$	$c_{d,\text{rear,suc}}$	$c_{l,\text{rear,pre}}$	$c_{d,\text{rear,pre}}$
REF	0.585	0.275	−1.958	0.365	2.543	−0.091
PTE1	0.541 ↓	0.282 ↑	−1.975 ↓	0.371 ↑	2.516 ↓	−0.090 ↓
PTE2	0.593 ↑	0.275	−1.953 ↑	0.365	2.546 ↑	−0.091

3 Supplementary section: Extended literature review on the relation between aerodynamic and aeroelastic phenomena in transonic flows

In the high-speed regime of commercial aircraft, transonic flow conditions are reached on the wings. This means that the local acceleration of the flow, which is induced by the airfoil geometry, yields a supersonic region in the leading part of the wing, which is terminated by a shock wave. When this flow field becomes unstable, the shock wave starts to oscillate along the chord of the airfoil, which is called transonic buffet. It usually occurs before the maximum lift is reached, which makes it an important feature for the aircraft design and flight envelope definition [6, 7]. This aerodynamic instability of the flow field can provoke a structural response, i.e., a motion of the wing's structure. Such aeroelastic phenomena constitute a hazard to flight safety since they can have an undesirable impact on the flight control system or even result in structural fatigue and material failure. The mechanisms of classical aeroelastic effects like flutter are well understood and depend on the freestream dynamic pressure [8]. In essence, these phenomena are governed by the interaction of elastic and inertial forces of the structure with unsteady aerodynamic forces. Since the latter substantially increase with aircraft speed, a critical flutter velocity can be defined, which indicates the onset of aeroelastic instability. In this case, the interaction of two structural modes, which are coupled via the flow, results in a self-excited structural vibration due to a structural mode becoming unstable. Thus, such critical flutter velocities must be excluded from the flight envelope [9].
However, there are also non-classical flutter problems which specifically occur in transonic flows. They possess different instability characteristics that also depend on the Mach number and the angle of attack. A well-known effect is the drop of the flutter boundary in the transonic regime, which is often called the transonic dip [10, 11] or double transonic dip for supercritial airfoils [12, 13]. Recent research has shown that transonic aeroelastic problems arise when the flow is close to or in the unsteady state of transonic buffet [14, 15, 16]. However, due to the immense computational cost associated with coupled fluid-structure interaction simulations, the majority of previous studies has analyzed aerodynamic and aeroelastic effects separately assuming that they are coupled only weakly. First, the aerodynamic loads induced by transonic buffet are calculated for a rigid wing. Second, these loads are imposed onto an elastic wing and the structural response is studied. Due to this classical decoupled approach, buffet is usually regarded as a fluid dynamics problem and most studies have focused on the aerodynamics of a rigid wing [17, 18, 19, 20]. Therefore, the fluid-structure interaction in buffet conditions is still not fully understood and since the physical mechanisms of non-classical transonic aeroelastic problems remain unclear, modern aircraft can still suffer from that [8].
So far, four conditions have been identified at which transonic aeroelastic effects occur [8]. Two types are related to pre-buffet conditions, i.e., close to buffet onset or offset, where the flow is stable but with a low stability margin such that it can be easily triggered. The other two conditions occur in unsteady flow, i.e.,

fully developed buffet flow. For the pre-buffet state, aeroelastic effects can either be induced by a structural or by a fluid dynamical instability. A prominent example for an instability in the structural mode is transonic buzz, also known as transonic single-degree-of-freedom (SDOF) flutter. In contrast to classical flutter, which is provoked by a coupling of structural modes at high dynamic pressure, the transonic SDOF flutter is induced by a coupling between a structural mode and a subcritical fluid mode [21]. Here, subcritical refers to a state where the flow has low damping like in pre-buffet conditions. A flow instability in pre-buffet conditions arises when considering an elastic wing, e.g., with a pitching degree of freedom. Compared to the aerodynamics with a rigid wing, premature buffet onset is observed. To be precise, at a constant Mach number, the angle of attack at which buffet onsets is reduced due to the interaction with the elastic structure [15]. Depending on the natural structural frequency, two instability patterns are observed. At high structural frequencies, an instability in the structural mode, essentially flutter, occurs. At low structural frequencies, forced vibration under buffet loads is induced. That means that the system frequency of the coupled fluid-structure system follows the buffet frequency of the uncoupled aerodynamic behavior. Similarly, the response amplitude is comparable to the forced vibration pattern [15].
When the unsteadiness associated with transonic buffet flow is fully present, two types of aeroelastic instabilities can occur. The structure can either oscillate under forced vibration, which is often called transonic buffeting [22, 23], or it can vibrate at the natural structural frequency, which is referred to as frequency lock-in [24, 25]. The first scenario can be studied conveniently with the decoupled flow/structural response approach outlined above. After determining the aerodynamic loads with the rigid wing, the aeroelastic response of the elastic wing is investigated. Due to this cheaper and less complex approach compared to directly considering the fluid-structure interaction, a lot of experimental and numerical studies have focused on this condition. However, a recent numerical study [14], which took the fluid-structure interaction into account, showed that the forced vibration effect does only occur for specific ratios of the natural structural frequency to the aerodynamic buffet frequency. For structural frequency values close to the buffet frequency or above (the upper limit is approx. 2.5 times the buffet frequency), frequency lock-in is observed. This phenomenon was previously noted for prescribed airfoil motion [24, 26] as well as aeroelastic responses [14, 25]. For example, Hartmann et al. [25] observed self-sustained heave/pitch oscillations close to the natural buffet frequency for an elastically suspended DRA 2303 airfoil at buffet conditions similar to the present study. Due to the energy transfer between fluid and structure, the amplitude of the shock wave oscillations, i.e., the spatial extent between the most upstream and most downstream shock wave location, is reduced by about 1 %. When the airfoil undergoes forced coupled heave/pitch oscillations at three frequencies close to the natural buffet frequency, the shock wave motion locks in with the respective excitation frequency. The authors showed that the aerodynamic feedback loop associated with the buffet phenomenon still exists in these scenarios.
Gao et al. [14] observed that the physical mechanism causing frequency lock-in in buffet conditions differs from the classical SDOF flutter. In the unstable buffet flow of the investigated NACA0012 airfoil, the coupling between a structural mode and a fluid mode causes frequency lock-in involving a different form of flutter compared to subcritical transonic flow conditions. Moreover, it was revealed that the structure oscillates with a higher amplitude compared to the forced vibration scenario. The authors also identified a transitional pattern referred to as veering, in which both frequencies, i.e., the structural frequency due to frequency lock-in and the buffet frequency induced by forced vibration, are observed in the system. This veering pattern occurs for structural frequencies close to the buffet frequency [14]. For the same airfoil geometry, Zhang et al. [27] investigated how buffet onset and offset during a flutter cycle affect the aeroelastic behavior. When the instantaneous pitching angle exceeds the buffet onset angle, the buffet flow provokes a decay of the structural motion. When the angle reduces below buffet onset, buffet decays and flutter inten-

sifies. The authors describe this coupled system as a nodal-shaped oscillation undergoing alternating phases of damping and diverging oscillation, which is different from the classical limit cycle oscillation flutter.
In one of the rare numerical studies focusing on 3D aerodynamic and structural phenomena, Houtman & Timme [28] investigated how including an elastic structure impacts the 3D aerodynamics of buffet. They demonstrated the importance of a coupled eigenmode solver in comparison to a conventional pk-type flutter method for detecting all relevant coupled modes. Similar to purely aerodynamic studies [29, 30], they identified the most dominant instability at the shock foot and its downstream separated boundary layer.
In summary, mainly three aeroelastic stability problems have been observed in transonic buffet conditions and flow conditions close to buffet onset and offset, in which the flow damping is low.

- **Forced vibration.** Due to the unsteady buffeting loads, i.e., the instability in the fluid flow, a forced structural response is provoked. The coupling frequency equals the uncoupled frequency of the aerodynamic buffet flow. The structural frequency is smaller or much larger than the buffet frequency. The vibration amplitude is rather low.

- **Frequency lock-in.** The aerodynamic instability excites structural vibrations, a form of transonic flutter, in which the coupling frequency corresponds to the natural structural frequency. The synchronization starts at structural frequencies similar to the buffet frequency and persists for structural frequencies up to about 2.5 times the buffet frequency. The oscillation amplitude is larger compared to the forced vibration resulting in a more severe threat to flight safety.

- **Veering.** A transitional state occurring for structural frequencies around the buffet frequency, in which forced vibration as well as frequency lock-in are observed.

Comparing the results of an analysis which considers a direct fluid-structure interaction to the findings based on the classical uncoupled approach, it was shown in [14] that uncoupled methods severely underestimate the amplitude of the structural vibrations by around one order of magnitude. Furthermore, the largest amplitude is predicted to occur at the resonance point, i.e., when the aerodynamic and the structural frequency synchronize, whereas a fluid-structure interaction analysis predicts the largest vibration at a structural frequency of about 1.5 times the buffet frequency. Moreover, a premature buffet onset was observed when the elasticity of the wing is taken into account [15]. All of these prediction mismatches could have a severe impact on the aircraft design process and require further investigations specifically focusing on the fluid-structure interaction in a unified framework.

References

[1] Rajendran, L., Zhang, J., Bane, S. & Vlachos, P. Uncertainty-based weighted least squares density integration for background-oriented schlieren. *Experiments in Fluids* **61**, 1–12 (2020).

[2] Ragni, D., Ashok, A., Van Oudheusden, B. & Scarano, F. Surface pressure and aerodynamic loads determination of a transonic airfoil based on particle image velocimetry. *Measurement Science and Technology* **20**, 074005 (2009).

[3] Ragni, D., Van Oudheusden, B. & Scarano, F. Non-intrusive aerodynamic loads analysis of an aircraft propeller blade. *Experiments in Fluids* **51**, 361–371 (2011).

[4] Feldhusen, A., Hartmann, A., Klaas, M. & Schröder, W. Impact of alternating trailing-edge noise on buffet flows. In *31st AIAA Applied Aerodynamics Conference*, 3028 (2013).

[5] Feldhusen-Hoffmann, A., Statnikov, V., Klaas, M. & Schröder, W. Investigation of shock–acoustic-wave interaction in transonic flow. *Experiments in Fluids* **59**, 1–13 (2018).

[6] Bendiksen, O. O. Review of unsteady transonic aerodynamics: Theory and applications. *Progress in Aerospace Sciences* **47**, 135–167 (2011).

[7] Breitenstein, C. Overview of criteria to estimate aerodynamic limits of the flight envelope of a transonic aircraft based on rans simulations. *CEAS Aeronautical Journal* **14**, 913–926 (2023).

[8] Gao, C. & Zhang, W. Transonic aeroelasticity: A new perspective from the fluid mode. *Progress in Aerospace Sciences* **113**, 100596 (2020).

[9] Tijdeman, H. & Seebass, R. Transonic flow past oscillating airfoils. *Annual Review of Fluid Mechanics* **12**, 181–222 (1980).

[10] Isogai, K. On the transonic-dip mechanism of flutter of a sweptback wing. *AIAA Journal* **17**, 793–795 (1979).

[11] Edwards, J. W., Bennett, R. M., Whitlow Jr, W. & Seidel, D. A. Time-marching transonic flutter solutions including angle-of-attack effects. *Journal of Aircraft* **20**, 899–906 (1983).

[12] Yates Jr, E. C., Wynne, E. C. & Farmer, M. G. Effects of angle of attack on transonic flutter of a supercritical wing. *Journal of Aircraft* **20**, 841–847 (1983).

[13] Miyake, T. & Terashima, H. Numerical investigation of double transonic dip behaviors in supercritical airfoil flutter. *AIAA Journal* **61**, 5365–5376 (2023).

[14] Gao, C. *et al.* Mechanism of frequency lock-in in transonic buffeting flow. *Journal of Fluid Mechanics* **818**, 528–561 (2017).

[15] Gao, C., Zhang, W. & Ye, Z. Reduction of transonic buffet onset for a wing with activated elasticity. *Aerospace Science and Technology* **77**, 670–676 (2018).

[16] Poplingher, L. & Raveh, D. E. Shock buffet and associated fluid–structure interactions of the benchmark supercritical wing. *AIAA Journal* **61**, 2381–2399 (2023).

[17] Lee, B. Oscillatory shock motion caused by transonic shock boundary-layer interaction. *AIAA Journal* **28**, 942–944 (1990).

[18] Giannelis, N. F., Vio, G. A. & Levinski, O. A review of recent developments in the understanding of transonic shock buffet. *Progress in Aerospace Sciences* **92**, 39–84 (2017).

[19] Accorinti, A., Baur, T., Scharnowski, S. & Kähler, C. J. Experimental investigation of transonic shock buffet on an OAT15A profile. *AIAA Journal* **60**, 6289–6300 (2022).

[20] Schauerte, C. J. & Schreyer, A.-M. Experimental analysis of transonic buffet conditions on a two-dimensional supercritical airfoil. *AIAA Journal* 1–17 (2023).

[21] Gao, C., Zhang, W. & Ye, Z. A new viewpoint on the mechanism of transonic single-degree-of-freedom flutter. *Aerospace Science and Technology* **52**, 144–156 (2016).

[22] Caruana, D., Mignosi, A., Corrège, M., Le Pourhiet, A. & Rodde, A. Buffet and buffeting control in transonic flow. *Aerospace Science and Technology* **9**, 605–616 (2005).

[23] Korthäuer, T., Accorinti, A., Scharnowski, S. & Kähler, C. J. Experimental investigation of transonic buffeting, frequency lock-in and their dependence on structural characteristics. *Journal of Fluids and Structures* **122**, 103975 (2023).

[24] Raveh, D. & Dowell, E. Frequency lock-in phenomenon for oscillating airfoils in buffeting flows. *Journal of Fluids and Structures* **27**, 89–104 (2011).

[25] Hartmann, A., Klaas, M. & Schröder, W. Coupled airfoil heave/pitch oscillations at buffet flow. *AIAA Journal* **51**, 1542–1552 (2013).

[26] Raveh, D. E. Numerical study of an oscillating airfoil in transonic buffeting flows. *AIAA Journal* **47**, 505–515 (2009).

[27] Zhang, W., Gao, C., Liu, Y., Ye, Z. & Jiang, Y. The interaction between flutter and buffet in transonic flow. *Nonlinear Dynamics* **82**, 1851–1865 (2015).

[28] Houtman, J. & Timme, S. Global stability analysis of elastic aircraft in edge-of-the-envelope flow. *Journal of Fluid Mechanics* **967**, A4 (2023).

[29] Paladini, E., Marquet, O., Sipp, D., Robinet, J.-C. & Dandois, J. Various approaches to determine active regions in an unstable global mode: application to transonic buffet. *Journal of Fluid Mechanics* **881**, 617–647 (2019).

[30] Iwatani, Y., Asada, H., Yeh, C.-A., Taira, K. & Kawai, S. Identifying the self-sustaining mechanisms of transonic airfoil buffet with resolvent analysis. *AIAA Journal* **61**, 2400–2411 (2023).
